# A Numerical Study of an Ellipsoidal Nanoparticles under High Vacuum Using the DSMC Method

**DOI:** 10.3390/mi14040778

**Published:** 2023-03-30

**Authors:** Jinwoo Jang, Youngwoo Son, Sanghwan Lee

**Affiliations:** Department of Mechanical Engineering, Hanyang University, 222 Wangsimni-ro, Seongdong-gu, Seoul 04763, Republic of Korea

**Keywords:** nanoparticle, DSMC, drag coefficient, ellipsoidal particle

## Abstract

The semiconductor and display manufacturing process requires high precision. Therefore, inside the equipment, fine impurity particles affect the yield rate of production. However, since most manufacturing processes are performed under high-vacuum conditions, it is difficult to estimate particle flow with conventional analytical tools. In this study, high-vacuum flow was analyzed using the direct simulation Monte Carlo (DSMC) method, and various forces acting on fine particles in a high-vacuum flow field were calculated. To compute the computationally intensive DSMC method, GPU-based computer unified device architecture (CUDA) technology was used. The force acting on the particles in the high-vacuum rarefied gas region was verified using the results of previous studies, and the results were derived for the difficult-to-experiment region. An ellipsoid shape with an aspect ratio rather than a spherical shape was also analyzed. The change in drag force according to various aspect ratios was analyzed and compared with the results of the spherical shape under the same flow conditions.

## 1. Introduction

Semiconductors and displays are essential components for most electronic products in use, such as laptops, tablet PCs, and smartphones. In addition, it is an electronic component necessary to implement artificial intelligence, the Internet of Things, drones, autonomous driving, and virtual reality, which are essential for the 4th industrial revolution.

Recently, semiconductors are manufactured by ultraprecise processes that constitute line widths of several nanometers or less. This ultraprecision process must be carried out in a high-vacuum state to minimize problems due to impurities. Ironically, however, within the high-vacuum environment, which is used to minimize impurities, it is also difficult to control small amounts of impurities. Therefore, it is essential to predict the movement of particles in a high-vacuum state.

Various investigators studied particle deposition on wafers by using experimental and numerical methods for several decades. The behavior of a spherical particle moving near a flat wall has been treated as a classical problem in fluid dynamics [1,2,3,4,5]. It studied the collision and sedimentation of particles on walls with no-slip boundary conditions. Furthermore, in the slip-flow regime, the movement of a particle close to a wall was investigated [6,7,8]. However, given the limitations of instrumentation and numerical methodologies, the calculation of nanoparticle deposition in a high vacuum is limited. Previous numerical methods to study the deposition of nanoparticles have assumed atmospheric pressure, room temperature, continuum assumptions, etc. In particular, particle deposition on wafers and photomasks mainly occurs under high vacuums and at high Knudsen numbers.

In this study, the motion of submicron particles is analyzed using the DSMC (Direct Simulation Monte Carlo) method in high-vacuum and free molecular systems. The DSMC method is a Lagrangian particle tracking simulation, which uses GPU (graphics processing unit) computing to calculate the particle’s trajectory by calculating the Stokes drag with slip correction.

DSMC can calculate fluid flow in a high vacuum outside of the continuum region and is widely used to predict supersonic gas flow [9]. The DSMC method is mainly based on the kinetic theory of gases, and the DSMC result approximates the solution of the Boltzmann equation as the number of representative particles to be calculated increases, the time interval, and cell size decrease [10,11,12]. In addition, DSMC not only models the rarefied gas region but also approximates the Navier–Stokes solution at very low Knudsen number limits [13,14,15]. DSMC has also been successfully validated in comparison with experimental and analytical results for shock waves in hypersonic applications [16,17,18]. The DSMC method has been accepted as one of the major numerical approaches for supersonic vehicle design and rarefied gas applications by improving the collision models [19,20], chemical reactions [21], ionization [22], radiation coupling [23], nanoporous material [24], and a hybrid technique with CFD [25]. However, this particle-based numerical approach is highly dependent on the performance of the computing hardware and requires many parallel-core machines. Therefore, GPU computing such as CUDA is essential for this study.

## 2. Theoretical Approach

### Drag Force in Slip Regime

Stokes’ law is a formula for calculating the force exerted by a fluid on an object. The resistance of the falling sphere is proportional to the diameter of the sphere, the velocity of the falling sphere, and the viscosity of the fluid as Equation (1) below,

(1) $$F_{D}=3\pi uvd_{p},$$

where $d_{p}$ is the particle diameter.

Stokes’ law describes the drag force ($F_{D}$) of a rigid particle by solving the Navier–Stokes equations for Reynolds numbers <1.0 and Knudsen numbers >1.0. An important assumption in Stokes’s law is that the relative velocity among the gases at the surface of the particle corresponds to zero. However, the assumption is not applicable for small particles with sizes approaching the mean free path of the gas. The drag force that actually acts on the particle is less than the drag force predicted by Stokes’s law when the particle size decreases. Therefore, under standard conditions, the error becomes significant for particles with a diameter of less than 1 μm. The use of a slip correction factor $C_{c}$ modifies Stokes’s law for applications with small particles to calculate the drag force. The expression is as follows Equation (2):

(2) $$F_{D}=\frac{3\pi uvd_{p}}{C_{c}}.$$

Numerous previous studies have been conducted to make the slip correction factor a function of the Knudsen number. By Cunningham, the drag force required to maintain the fluid velocity at a high Knudsen number and the slip correction factor were derived in the form of $\left(1+AKn\right)$, and the study of parameter A started.

The slip correction factor was first derived as Equation (3) below [26]:

(3) $$C_{c}=1+\frac{2.52\lambda }{d_{p}}=1+1.26\;Kn,$$

where *λ* is the mean free path and Kn is the Knudsen number.

Around the same time, Millikan [27] experimentally confirmed a linear increase in the correction term for the mean free path in the Cunningham formulation for Knudsen numbers less than 0.3. In addition, Weber [28] expressed parameter A (Equations (4) and (5)) as a function of the Knudsen number through an experimental method.

(4) $$C_{c}=1+A\cdot Kn,$$

(5) A=α+β·exp(−γ/Kn).

Here, *α*, *β*, and *γ* are experimentally obtained constants. Since then, many experimental studies have been conducted to find *α*, *β*, and *γ* to represent parameter A in the study of the slip correction factor.

The air viscosity of $\mu_{23}=1.824\times 10^{-5}\;{\mathrm{kg}\;m}^{-1}{\;s}^{-1}$ was used for slip correction experiments given an average of the most accurate measurements taken in the early 1900s [29].

The corresponding expression is as follows in Equation (6):

(6) $$C_{c}=1+Kn\left[1.246+0.42e^{\frac{-0.87}{Kn}}\right].$$

The weighted average value of the viscosity of air as $\mu_{23}=\left(1.83245\pm 0.00069\right)\times 10^{-5}\;\mathrm{kg}/\mathrm{ms}$ based on six different results was suggested [30], and this involved correcting for temperature by using the Sutherland Equation (7) as follows:

(7) $$\mu_{T}=\mu_{23}{\left(\frac{T}{T_{0}}\right)}^{1.5}\frac{\left(T_{0}+110.4\right)}{\left(T+110.4\right)}.$$

It is not possible to directly measure the mean free path of air, *λ*, although this is defined from the kinetic theory relationship for viscosity as in the following Equation (8):

(8) $$\lambda =\lambda_{0}\left(\frac{T}{T_{0}}\right)\left(\frac{P}{P_{0}}\right)\frac{\left(1+\frac{110.4}{T_{0}}\right)}{\left(1+\frac{110.4}{T}\right)},$$

where $\lambda_{0}$ is 67.3 nm which is the mean free path of Sutherland’s formula.

The slip correction factor at reduced pressures and high Knudsen number was experimentally investigated by using polystyrene latex particles [31].

They employed nano differential mobility analyzers to define the slip correction factor by measuring the electrical mobility for particle sizes corresponding to 100.7 nm, 269 nm, and 19.90 nm as a function of pressure.

As a result, a modified slip correction factor was suggested in the following Equation (9) [31]:

(9) $$C_{c}=1+Kn\left[1.165+0.483e^{\frac{-0.997}{Kn}}\right]\left[0.5<Kn<83\right].$$

## 3. Numerical Methods

### 3.1. Direct Simulation Monte Calro

General flow analysis was performed using the Navier–Stokes equation; however, in the rarefied gas domain, the continuum assumption could not be applied to general fluid flow analysis. In continuum fluid dynamics, the motion of a liquid or gaseous object is described as a continuous medium, ignoring the fact that it consists of molecules. Because the mean free path *λ* of gas molecules is very short (several nanometers to micrometers) in the gas flow from the viewpoint of continuum dynamics, molecular flow can be represented by the viscosity coefficient for the momentum exchange in intermolecular collisions.

However, as the gas becomes leaner, the mean free path becomes longer, the continuous properties of these fluids are lost, and the Navier–Stokes equation loses its meaning. The assumption of a fluid continuum under gas-lean conditions can be distinguished using the Knudsen number (Kn). The Knudsen number is one of the dimensionless quantities expressing the characteristics of flow. It is a numerical value that expresses the mutual relationship between gas and particles. It refers to the value obtained by dividing the mean free path (*λ*) by the characteristic length (*L*) as in Equation (10) below:

(10) $$Kn=\frac{\lambda }{L}.$$

The Navier–Stokes equation error can start to occur when the Knudsen number is larger than 0.1, and because the continuum characteristic disappears in the rare-gas region with a Kn number larger than 10, the flow should be analyzed using the Boltzmann equation.

DSMC is one of the methods used to numerically solve the Boltzmann equation. It performs analyses by applying a probabilistic model to overcome the problems caused by a large number of analysis domains to calculate every molecule or those caused by a very long analysis time.

DSMC replaces a certain number of molecules with one representative particle (F_N_, 10^11^–10^15^). It approximates the physical phenomenon represented by the Boltzmann equation using a statistical method. In the DSMC method, flow is caused by repeating movement, collision, deposition, and chemical reactions using these representative particles, and various characteristics such as velocity, temperature, and pressure can be expressed through statistical techniques. The collision algorithms are important in DSMC methods and calculate the most complex terms of the Boltzmann or Kac probability equations. The DSMC schemes can be grouped into two groups with respect to collision treatment [32]. The first group is based on the Boltzmann equation and includes TC [33], NTC [34], NC [35], and MFS [36] methods. The second group is based on the Kac stochastic equation, and there are BT [37] and SBT [38,39] methods. In this study, we used the NTC collision scheme, which is the most widely used.

**Figure 1:**
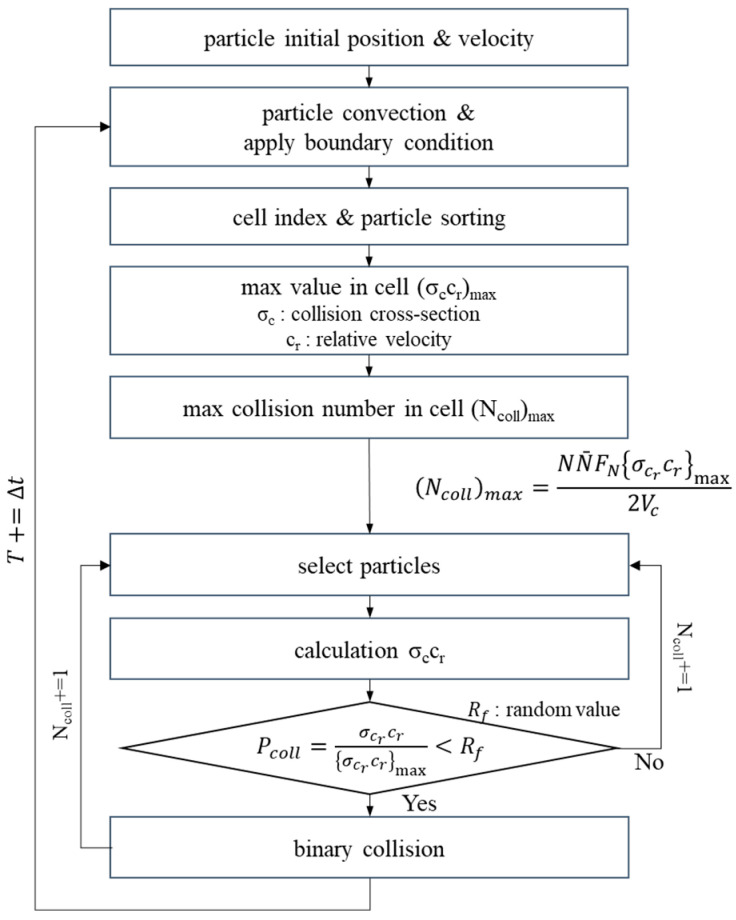
DSMC algorithm process.

The DSMC algorithm for flow analysis in a high-vacuum environment is summarized in Figure 1 as follows:

(1) Determine the position of particles in the initial analysis area. The number of particles is set by adjusting the F_N_ value according to the temperature and pressure conditions in the initial flow region. It also assigns an initial velocity to every particle for a given initial temperature. At this time, the magnitude of the velocity is determined according to the Maxwell–Boltzmann distribution.

(2) Each particle moves according to a given speed and time step. At this time, the given boundary condition is applied by determining the collision with the wall, inlet, and outlet.

(3) Sort the particles included in each cell by the cell index according to the position of each particle in the cell unit divided by the flow space.

(4) Find the product of the relative velocity and the collision cross-sectional area between all particles included in each cell and store the maximum value of this value.

(5) Calculate the maximum number of collisions in each cell using the maximum value of the product of the relative velocity and the collision cross-sectional area. A collision pair is randomly selected within the cell as many as the number of collisions obtained, and the actual collision is calculated according to the probability distribution.

### 3.2. GPU Computing (CUDA)

The GPU was developed to use a parallel computation structure specialized for the real-time processing of large amounts of data. Unlike CPUs with a small number of high-performance cores, GPUs have hundreds to thousands of small and fine cores per chip, and through this, a large amount of data are distributed and processed, resulting in a tremendous performance compared to CPUs.

GPU technology has been utilized not only for image processing but also in other fields, and the form used by early developers is called GPGPU. At that time, it was used by very few developers and was a very difficult advanced technology.

The compute unified device architecture (CUDA), released by Nvidia in 2006, is a computing platform that can utilize GPU. CUDA greatly improves access to GPU resources by making it a high-level language and syntax familiar to general developers such as C/C++. It can be used in many different fields.

In terms of computational performance, the analysis performance is tens to hundreds of times faster than that of CPUs of similar prices, but it does not show high performance in all fields. The structure of the GPU has numerous iterative calculations; therefore, the GPU shows great performance in areas where performance can be improved through parallel processing.

Recently, GPUs are being used in fields such as graphics and deep learning; they have also been widely employed in industries such as autonomous vehicles, AI technology development, and virtual currency mining.

In the case of particle-based flow analysis methods such as DSMC, each particle behaves independently according to the surrounding environment, and it is a suitable method for parallel processing calculations. Therefore, in this study, a DSMC program was developed and was based on a GPU.

## 4. Results and Discussion

### 4.1. Microchannel Flow

In order to validate the DSMC code used in this study, numerical results and analytic solutions were compared in microchannel flow. The geometry of the microchannel flow was 1 μm in channel height and 5 μm in length and the pressure was calculated using the conditions in Table 1.

The gas used for DSMC calculation was argon gas at 273 K, and the total number of particles was 200,000. The wall boundary condition of the microchannel was a temperature of 273 K. The theoretical solution for the Poiseuille flow in the microchannel can be obtained from the solution of the Navier–Stokes equation using the slip velocity boundary condition. The theoretical solution was defined using the pressure described in Equation (11) and the velocity described in Equation (12), and the numerical solution was compared by the normalized value based on the maximum velocity at 1/2 of the microchannel [40].

In Equation (11), *P*(*x*) is the pressure, Kn is the Knudsen number, $Kn_{0}$ is the Knudsen number at the outlet, and *σ* is the tangential accommodation coefficient. The analytical expression of velocity distribution *u* is given by Equation (12) below, where *μ* is the viscosity, and *H* is the channel height:

(11) $$P\left(x\right)=-6\sigma Kn_{0}+\sqrt{{\left(6\sigma Kn_{0}\right)}^{2}+\left(P^{2}+12\sigma Kn_{0}P\right)\left(1-\frac{x}{L}\right)+\left(1+12\sigma Kn_{0}\right)\frac{x}{L}},$$

(12) $$u=\frac{H^{2}}{2\mu }\frac{dP\left(x\right)}{dx}\left[\frac{y^{2}}{H^{2}}-\frac{y}{H}-\sigma Kn\right].$$

**Figure 2:**
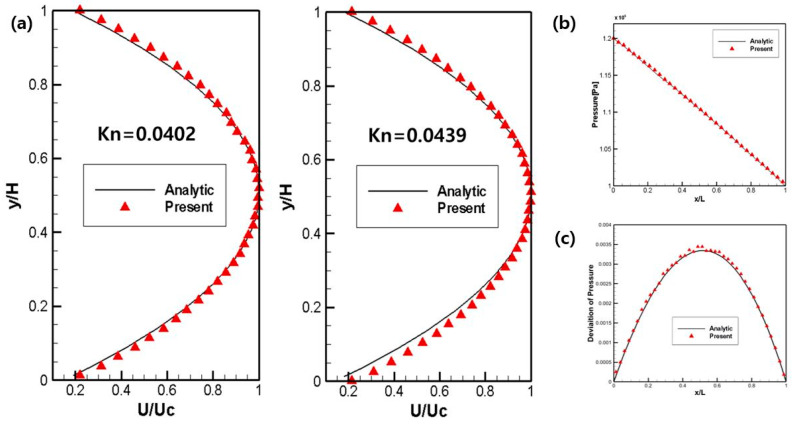
(**a**) Velocity profiles for microchannel flow. (**b**) Pressure profile (Kn = 0.0439) of microchannel flow. (**c**) Deviation in the pressure (Kn = 0.0439) of microchannel flow.

As shown in Figure 2a,b, the results of the numerical analysis of pressure and velocity of the DSMC method used in this study were verified to be in good agreement with the theoretical results of Equations (11) and (12). In addition, the time-averaged steady-state flow field vector result with the use of a large number of particles and sufficient sampling process is shown in Figure 2c, which also agrees well with the theoretical solution.

### 4.2. Nanoparticle Drag Force

In this study, a validated DSMC algorithm was used to calculate the drag force acting on a fine particle in a high vacuum, and the particle diameter size used in the calculation ranges from several hundred nanometers to several micrometers. To model the uniform flow field around the fine particles, a cylinder channel that is sufficiently larger than the particle diameter was used, and the flow field was constructed by filling the inside of the cylinder with air molecules. In addition, by applying a no-slip boundary condition between the cylinder channel wall and the DSMC particles and a velocity boundary condition on the cylinder channel wall, it was possible to create a flow field with a uniform velocity around the fine particles.

**Figure 3:**
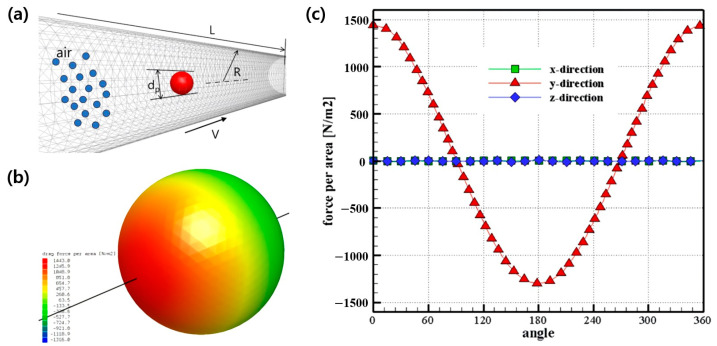
(**a**) Schematic diagram of the calculation domain for the drag force. (**b**) Force-per-area contour on the particle surface. (**c**) Force per area on particle surface for various angles.

The direction of the uniform flow field becomes the axial direction of the cylinder channel, and the fine particles inside the cylinder channel are fixed so that the drag force according to the flow can be calculated. Figure 3a shows a cylinder channel with a flow field and fixed microparticles.

The air molecular mass and diameter are $4.815\times 10^{-26}\;\mathrm{kg}$ and $3.7\times 10^{-10}\;m$, respectively. The calculation domain consists of 20×20×200 cells. The number of representative particles per cell is 40, which is more than the minimum number of particles suggested in a previous study [41]. The total number of representative particles for DSMC calculation in the cylinder channel volume is about 3 million.

The initial velocity of the air particles was calculated for a velocity distribution corresponding to a temperature of 300 K. The velocity distribution according to temperature was defined by Maxwell–Boltzmann distribution, and the RMS velocity and number density distribution about velocity were described in Equations (13) and (14) below:

(13) $$v_{rms}=\sqrt{\int_{0}^{\infty }v^{2}f\left(v\right)dv}=\sqrt{\frac{3k_{B}T}{m}}=\sqrt{\frac{3RT}{M}},$$

(14) $$f\left(v\right)=4\pi {\left(\frac{m}{2\pi k_{B}T}\right)}^{3/2}v^{2}exp\left[\frac{-mv^{2}}{2\pi k_{B}T}\right].$$

When the air particles inside the cylinder channel collide with a wall having a constant temperature, it is converted into a velocity corresponding to the wall temperature according to the diffuse reflection coefficient. Given these conditions, the wall moves at a constant speed along the axis of the cylinder channel and a spherical fine particle under the force is fixed at the center of the axis of the cylinder channel, and the drag force is calculated.

To calculate the drag force of the particle according to the Knudsen number, various flow conditions were used. Table 2 shows the pressure according to the Knudsen number in the analysis domain. In these cases, the particle size was 100 nm, and the temperature was fixed at 300 K.

The force acting on a spherical particle in the center of the cylinder is determined from the rates of delivery, and the removal of momentum and molecules are reflected from the particle surface within time intervals ${\Delta t}_{s}$ following Equation (15):

(15) $$F_{D}=\frac{1}{\Delta t_{s}}\sum m\left(V_{x}^{\left(+\right)}-V_{x}^{\left(-\right)}\right),\;\Delta t_{s}=N_{iteration}\Delta t,$$

where Δ*t* is a time step, $N_{iteration}$ is the iteration number corresponding to a time interval, m is the mass of the external fluid, $V_{x}^{\left(+\right)}$ is the velocity before the collision with the spherical particle, and $V_{x}^{\left(-\right)}$ is the velocity after collision. The force components in each direction were obtained.

In this study, the total analysis time was 50 µs. The time step was about 0.05 ns, and analysis was performed over about 1 million iterations. This analysis was computed using the GPU. The GPU used for the analysis was an NVIDIA GTX980, and the analysis time was 48 to 96 h. To obtain the fine-particle drag force, statistical processing was performed for the total analysis time to obtain the time average value.

The drag force was calculated from the collision between the DSMC air particles and the fine particle, and Figure 3b shows the force generated in the flow direction on the surface of the fine particle. Figure 3c shows the magnitude of the force according to the position for each angle. The fine particle was divided at an angle of θ based on the opposite direction of flow, and the calculated force was expressed as a graph. The force applied to the surface of the fine particle in the flow direction was assumed to be a positive value.

As shown in Figure 3c, it can be confirmed that the force generated in the flow direction according to the angle was calculated. However, it can be seen that the force in the direction perpendicular to the flow direction was calculated relatively close to zero. In this study, the drag force, considering the slip correction factor, was validated by comparing the results of experimental data with velocities of 10 m/s and 50 m/s.

The results of previous experimental studies on drag force were up to a Knudsen number of 83. In addition, previous numerical analysis studies on drag had difficulties in predicting the deposition of particles in the high-vacuum region due to the limitation that the drag force was calculated only in the continuum region.

However, the drag force was calculated using the DSMC method, as shown in Table 2, at a temperature of 300 K and a high-vacuum pressure of 13,851 Pa to 14 Pa, and up to a Knudsen number of 10,000. The calculation domain is illustrated in Figure 3a, and the pipe radius (R) was set to be 50 times the spherical particle size (*d_p_*). 

**Figure 4:**
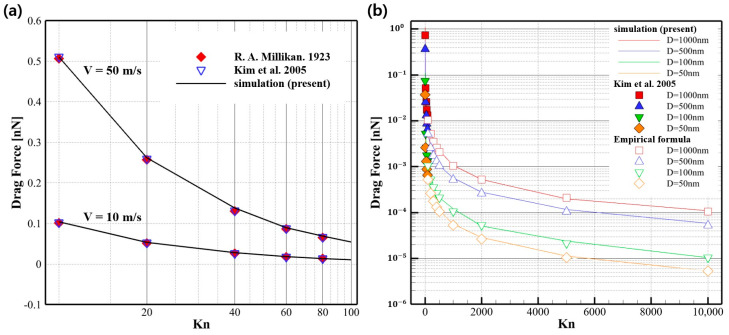
(**a**) Drag force for various values of Kn at velocities of 10 m/s and 50 m/s [29,31]. (**b**) Drag force for various particle sizes [31].

Figure 4a shows the drag force acting on the particle for each Knudsen number at 10 m/s and 50 m/s, respectively. The results for Knudsen numbers ranging from 10 to 80 are in good agreement for the two experiments. These results used a spherical particle with a diameter of 100 nm to calculate the drag force.

The simulation results in Figure 4b show drag force up to a Knudsen number of 10,000 for particles of various sizes less than 1 μm. The force was calculated by applying spherical particles in the range of 50 nm to 1000 nm, which corresponds to the particle size that causes defects in wafers and photomasks in semiconductor manufacturing. The simulations were calculated by varying the pressure in the system to match the Knudsen number for each particle size. 

In previous studies [29,31], the drag force received by particles in a high-vacuum state was only tested in the region below a Knudsen number of 83, and the experimental results in the region higher than a Knudsen number of 83 were very insufficient. To calculate the drag force at a high Knudsen number, the drag force of spherical particles of various sizes in a known region (*Kn* < 83) was first obtained by using the DSMC algorithm, and the DSMC results were then verified with the empirical formula with the slip correction factor described in Equation (10). In addition, areas of Knudsen number 100 or higher, which are difficult to implement through experiments, were derived as a result of DSMC code analysis. As a result, as shown in Figure 4b, the drag force of spherical particles under a Knudsen number 10,000 was in good agreement with the results obtained by numerical simulation and the empirical formula.

### 4.3. Drag Force of Ellipsoid Particle

**Figure 5:**
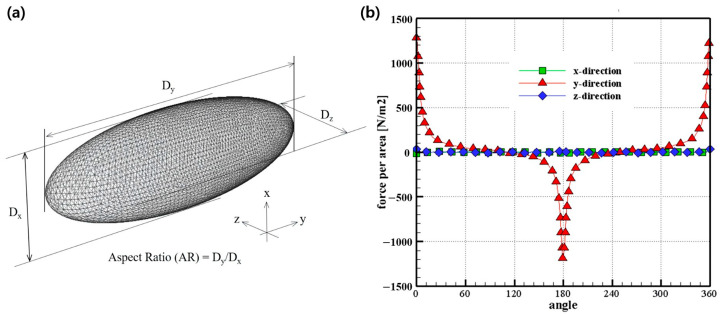
(**a**) The shape of the ellipsoid with aspect ratio. (**b**) Force per area on ellipsoidal particle surface for various angles.

In the previous section, the shape of the particle was modeled as a sphere, and the force was calculated for various Kn and particle diameters in the high-vacuum region. However, in reality, the shapes of most nanoscale particles are not perfectly spherical. Therefore, in this section, the shape of the particle is modeled as an ellipsoid, as shown in Figure 5a, and the drag forces are analyzed for various ratios of horizontal to vertical. In Figure 5a, the distances of three-dimensional directions are described as *D_x_*, *D_y_*, and *D_z_*, which are in the x, y, and z-directions, respectively.

The flow is in the y-direction, and the lengths in the x- and z-directions perpendicular to the flow direction have the same value. The ratio of the length in the flow direction to the length in the direction perpendicular is called the aspect ratio (AR). In this study, the drag force was calculated by changing the length ratio from 1 to 10.

When the AR is 1, it has the shape of a sphere analyzed previously, and when it is 10, it has a shape that elongates in the flow direction, but the projection shape in the flow direction has a circular shape with a diameter of *D_z_*. In addition, to determine the effect of the diameter of the fine particles, the vertical diameter *D_x_* was analyzed at 50 nm and 100 nm.

Figure 5b shows the values of the drag force measured on the ellipsoid surface with respect to the flow direction. It can be seen that, unlike the results in the previous sphere shape, the drag force is almost measured on the front and back surfaces of the ellipsoid microparticles. This indicates that the drag force in the rare-gas region is dominated by the effect of the direct collision rather than by the force caused by friction with other molecules.

**Figure 6:**
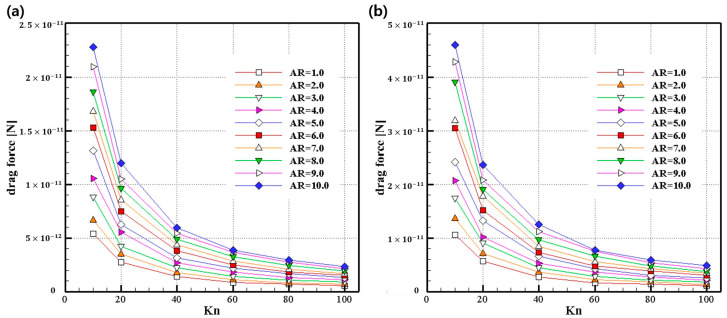
(**a**) Drag force for various aspect ratios ($D_{x}=50\;\mathrm{nm}$). (**b**) Drag force for various aspect ratios ($D_{x}=100\;\mathrm{nm}$).

Figure 6 shows the drag force profile for two different diameters (50 nm and 100 nm), which have various ARs. An AR = 1 means that the shape of the particle is spherical, and AR = 2 to AR = 10 means that the horizontal length increases, and thus the shape of the particle becomes flatter. As a result, a high AR leads to an increase in the drag force.

The trend of the drag force for the difference between Kn and AR with different diameters is similar. As Kn increases, the drag force decreases, and as AR increases, the drag force increases under the same flow conditions.

**Figure 7:**
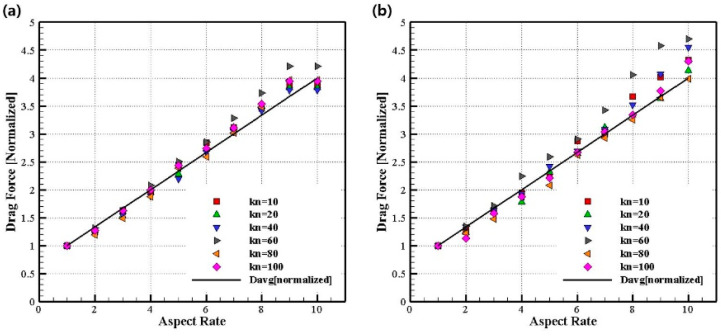
(**a**) Normalized drag force according to various aspect ratios ($D_{x}=50\;\mathrm{nm}$). (**b**) Normalized drag force according to various aspect ratios ($D_{x}=100\;\mathrm{nm}$).

We calculated the ratio of increase in the drag force as the AR increases. Figure 7 shows the ratio of the drag force based on the sphere-shaped drag force with AR = 1 as AR increases when Kn is the same. The drag force ratio can be expressed using the following formula:

(16) $$F_{D}/F_{D,\;AR=1}.$$

The ellipsoid was constructed by increasing the length in the flow direction based on spheres with diameters of 50 and 100 nm. When AR was 1, the ratio of the average length as AR increased based on the average length was calculated. As AR increases, the average length along the three axes also linearly increases, and the ratio of the average length is shown in Figure 7, which can be expressed by the following formula.

(17) $$D_{avg}=\left(D_{x}+D_{y}+D_{z}\right)/3,\;ratio=D_{avg}/D_{avg,\;AR=1}.$$

As shown in Figure 7, the ratio of drag force linearly increases with AR, and it is confirmed that the ratio is independent of the number of Kn. In addition, it can be confirmed that the increase in width is almost identical to the ratio of the average length.

## 5. Conclusions

The semiconductor-display manufacturing process, which requires high precision, is mainly performed in a high-vacuum environment. In ultrafine processes, impurities with a size of several tens of nanometers cause serious problems in the manufacturing process. Therefore, in this study, the force applied to fine particles for flow in a high vacuum was analyzed.

In this study, to first verify the DSMC code, the flow in a generally well-known microchannel was analyzed. The computational analysis results were compared with the theoretically obtained exact solution, which also agrees well with the theoretical solution.

The flow field was modeled using the DSMC technique to create a flow in a high-vacuum environment, and a GPU-based DSMC program was developed and analyzed on a high-performance GPU using CUDA.

Analysis was performed for Knudsen numbers ranging from 100 to 10,000 and various particle sizes. This study was performed in an area that could not be performed as an experiment in previous studies. It was confirmed that the drag force obtained in this study agrees well with the results predicted by the existing empirical formula.

The results of this study can be used as a basis for controlling fine particles through flow in various processes in a high-vacuum environment.

## Figures and Tables

**Table 1:** Simulation cases for the channel flow.

Case	Inlet Pressure [Pa]	Outlet Pressure [Pa]	Kn
1	$1.4\times 10^{5}$	$1.0\times 10^{5}$	0.0402
2	$1.2\times 10^{5}$	$1.0\times 10^{5}$	0.0439

**Table 2:** Pressure for Knudsen number [$d_{p}=100\;\mathrm{nm},\;T=300\;K]$.

Knudsen Number	Pressure [Pa]
10	13,851
20	6925
50	2770
100	1385
500	277
1000	139
5000	28
10,000	14

## Data Availability

All of the data are available upon request to the corresponding author.
